# New fat free mass - fat mass model for use in physiological energy balance equations

**DOI:** 10.1186/1743-7075-7-39

**Published:** 2010-05-09

**Authors:** Diana Thomas, Sai Krupa Das, James A Levine, Corby K Martin, Laurel Mayer, Andrew McDougall, Boyd J Strauss, Steven B Heymsfield

**Affiliations:** 1 Department of Mathematical Sciences, Montclair State University, Montclair, NJ, USA; 2 Energy Metabolism Laboratory, Jean Mayer USDA Human Nutrition Center at Tufts University, Boston, MA, USA; 3 Department of Medicine, Endocrine Research Unit, Mayo Clinic and Mayo Foundation, Rochester, MN, USA; 4 Ingestive Behavior Laboratory, Pennington Biomedical Research Center, Baton Rouge, LA, USA; 5 Columbia University, College of Physicians and Surgeons, The New York State Psychiatric Institute, New York City, NY, USA; 6 Department of Mathematical Sciences, Montclair State University, Montclair, NJ, USA; 7 Monash University Department of Medicine, Monash Medical Centre, Clayton, Victoria, Australia; 8 Merck & Co, Rahway, NJ, USA

## Abstract

**Background:**

The Forbes equation relating fat-free mass (*FFM*) to fat mass (*FM*) has been used to predict longitudinal changes in *FFM *during weight change but has important limitations when paired with a one dimensional energy balance differential equation. Direct use of the Forbes model within a one dimensional energy balance differential equation requires calibration of a translate parameter for the specific population under study. Comparison of translates to a representative sample of the US population indicate that this parameter is a reflection of age, height, race and gender effects.

**Results:**

We developed a class of fourth order polynomial equations relating *FFM *to *FM *that consider age, height, race and gender as covariates eliminating the need to calibrate a parameter to baseline subject data while providing meaningful individual estimates of *FFM*. Moreover, the intercepts of these polynomial equations are nonnegative and are consistent with observations of very low *FM *measured during a severe Somali famine. The models preserve the predictive power of the Forbes model for changes in body composition when compared to results from several longitudinal weight change studies.

**Conclusions:**

The newly developed *FFM*-*FM *models provide new opportunities to compare individuals undergoing weight change to subjects in energy balance, analyze body composition for individual parameters, and predict body composition during weight change when pairing with energy balance differential equations.

## Background

Modeling body weight regulation, and thus energy balance, involves quantifying appropriate system inputs, outputs, and balances. Mathematical models based on the energy balance equation provide descriptions of the impact of physiological changes and quantitative predictions of body mass during weight change. The development of energy balance models can have two approaches: 1. descriptions of the impact of physiological changes 2. quantitative predictions of body mass during weight change applying minimal individual baseline information.

The Hall model [1] is developed around the first approach where a system of five differential equations is carefully determined to reflect the specific flow of macronutrient energy during weight change. Although the model is physically descriptive, simulations require information of the macronutrient composition of individual baseline (zero energy balance) and target energy intake (change in energy intake by caloric restriction or overfeeding) along with numerous parameter estimates.

On the other hand, several models have been developed which require less baseline and parameter estimation [2-8]. The simplified models share several characteristics which reduce the number of state variables and equations. The first major simplification assumes glucose/glycogen mass is modeled by a time averaged constant, thereby eliminating the carbohydrate balance equation and its associated parameters and baseline inputs [3].

The second major simplification assumes that fat-free mass (*FFM*) can be written as a pre-defined function of fat mass (*FM*), eliminating the protein balance equation and the *FFM *state equation. The choice of function varies depending on the selected model, however, most functions are linear functions of *FM *[2,4-8]. Recently, the Forbes model was successfully incorporated into a two dimensional differential equation with satisfactory results [3]. A reduced one dimensional equation was consequently derived (Equation 25 in [3]).

The cross-sectional Forbes model was developed using group mean data of 167 women (Equation 1, Table 1). A key feature of Forbes model is that the developed cross-sectional function is concave and this observation supports a long-held view that longitudinal changes in body mass at the lower biological range are associated with very large changes in *FFM *while fat loss at the upper range has only minimal *FFM *effects. Forbes and others subsequently collected various study data to test his model in a longitudinal context and the results appeared satisfactory in predicting *FFM *changes for any given change in *FM *[9-12].

**Table 1:** List of Formulas

Number	Formula Reference and Citation	Equation
1	Forbes Equation (Females) [10]	*FFM *= 10.4 ln(*FM*) + 14.2

2	Differential Equation Energy Balance Model [3]	

3	Generalized Forbes Equation [3] Females [10]	*FFM *= 10.4 ln(*FM *= D)

4	Forbes Equation (Males)	*FFM *= 13.8 ln(*FM*) + 16.9

These satisfactory results provide an attractive choice for a one dimensional energy balance differential equation model (Equation 2, Table 1), where *c*_*l *_and *c*_*f *_are the energy conversion constants for one kg of *FFM *and *FM*, respectively, and *E *represents the rate of energy expended in kcal/d and is a function of *FM *and *FFM *[3]. Because the Forbes model is not valid for low *FM *we propose a new *FFM *- *FM *model which corresponds to experimental data on low *FM *while still preserving the predictive properties of the Forbes model.

A second consideration in applying the Forbes model within a one dimensional differential equation is that longitudinal body composition data does not actually "travel" down the Forbes curve. Body composition travels down a parallel translation of the original Forbes curve [12]. This does not affect estimates of Δ*FFM *as the slope of two parallel curves is identical. As past applications of the Forbes model were only concerned with estimating Δ*FFM*, identifying which translate of the Forbes model longitudinal body composition data traveled was not a concern. Because, in the one dimensional model, the rate of energy expenditures is dependent on the precise quantity of *FFM *at any given time during weight change, the specific translate of the Forbes curve must be derived. As a result, each individual simulation of the differential equation requires recalculation of a calibration parameter, D [3] (Equation 3, Table 1).

We formulate a new class of fourth order polynomial models using the newly released National Health and Nutrition Examination Survey (NHANES) data (*n *= 11, 186). We show that the intercepts of these polynomial equations reflects real-life body composition through comparison to data from the recent severe famine in Somalia [13]. We also show that longitudinal data travels the trend set by NHANES and that the new models predict the changes in *FFM *with equal accuracy to the Forbes model, thereby preserving the most descriptive conclusions derived from the Forbes model. The developed NHANES *FFM*-*FM *models provide new opportunities to compare individuals undergoing weight change to subjects in energy balance, analyze body composition for individual parameters, and predict body composition during weight change by pairing with energy balance differential equations.

## Methods

### Experimental Design and Rationale

An existing large database reflective of the United States non-institutionalized population was used to determine cross-sectional relationships between *FFM *and *FM *considering age, height, race and gender as additional covariates. We used the cross-sectional models to answer three main questions related to pairing a *FFM *model to an energy balance equation: Do the cross-sectional models preserve the accuracy of predictions provided by the Forbes model for changes in *FFM *during weight change? Do the cross-sectional models have a non-negative intercept that reflects observed data? How do the cross-sectional models vary with age, height, gender and race? To answer these questions we analyzed two existing cross-sectional body composition databases consisting of athletes and subjects with anorexia nervosa and five existing weight change databases which reflect changes in body composition due to caloric restriction, caloric restriction combined with exercise, gastric bypass surgery, laparoscopic adjustable gastric band surgery, and overfeeding.

### Subjects and Measurements

All of the following studies were approved by respective Institutional Review Boards and subjects provided written informed consent prior to participation.

#### Cross Sectional Data

NHANES is a program designed to assess the health and nutritional status of adults and children in the United States. NHANES performs a continuous, nationally representative health survey of the civilian, non-institutionalized United States population, collecting data on about 5000 persons each year from interviews, physical examinations, and medical tests including bone densitometry. In 1999 NHANES began performing dual-energy x-ray absorptiometry whole body measurements on survey subjects 8 years old and older in three mobile examination centers. Our study focus is on the adult age, gender, height and ethnicity-specific DXA body composition reference database developed from the NHANES survey data collected from 1999 to 2004. We expressed *FFM *and FM as indices to height (i.e., *FFM *and fat/height^2 ^(kg/m^2^)) as reported by [14-16], referred to as FFMI and FMI, respectively (Table 2).

**Table 2:** Baseline Characteristics of Study Cohorts

Study	*n*		Age (yrs)		Height (cm)	
	M	F	M	F	M	F
NHANES [14,15]	5617	5566	46.5 ± 20.0	47.5 ± 20.1	174.3 ± 7.9	160.6 ± 7.2

Anorexia [17]	--	38	--	26.0 ± 5.8	--	162.7 ± 6.0

Athletes [18]	62	62	31.9 ± 10.5	35.7 ± 10.6	177 ± 7.3	164.8 ± 6.3

Bariatric Surgery [20,21]	--	17	--	41.7 ± 7.4	--	165.2 ± 6.3

CALERIE [19]	15	20	38.0 ± 7.0	37.6 ± 5.5	178.1 ± 7.0	165.7 ± 6.6

Gastric Bypass Surgery [22]	--	24	--	38.7 ± 9.4	--	163.0 ± 7.0

Overfeeding [24,25]	10	12	34.9 ± 8.1	40.8 ± 6.7	177.0 ± 3.9	164.6 ± 6.0

Minnesota [23]	30	--	--	--	179.4 ± 5.4	--

	**Total Body Mass (kg)**		**Fat Mass (kg)**		***FFM *(kg)**	
	**M**	**F**	**M**	**F**	**M**	**F**

NHANES [14,15]	83.7 ± 19.0	73.5 ± 19.3	24.2 ± 10.4	30.6 ± 12.3	60.3 ± 10.4	43.5 ± 8.2

Anorexia [17]	--	41.9 ± 5.9	--	4.9 ± 3.4	--	37.0 ± 4.8

Athletes [18]	79.3 ± 13.2	60.1 ± 9.0	11.9 ± 7.4	13.2 ± 6.1	67.4 ± 9.0	46.8 ± 5.4

Bariatric Surgery [20,21]	--	91.9 ± 9.6	--	45.3 ± 7.0	--	46.6 ± 4.3

CALERIE [19]	90.0 ± 4.0	76.8 ± 7.8	22.0 ± 4.0	29.3 ± 5.4	68.0 ± 7.3	47.5 ± 4.2

Gastric Bypass Surgery [22]	--	131.0 ± 8.0	--	70.7 ± 19.5	--	60.3 ± 10.1

Overfeeding [24,25]	88.0 ± 19.5	78.7 ± 19.5	27.6 ± 15.2	32.3 ± 15.3	60.4 ± 7.2	46.4 ± 5.5

Minnesota [23]	70.7 ± 5.9	--	10.3 ± 4.1	--	60.4 ± 4.4	--

Group values are mean ± SD.

Cross sectional body composition data for women with anorexia nervosa between the ages of 18 and 45 years was obtained from the study described in [17]. Subjects were patients receiving treatment at the Eating Disorder Research Unit at the New York State Psychiatric Institute (NYSPI), Columbia University Medical Center (CUMC). Several different types of body composition methods were applied in [17] and we focus our report on the DXA measurements (Table 2).

Cross sectional DXA body composition data was collected at the New York Obesity Research Center on 124 healthy adults actively participating in exercise training programs including body building, cycling, and long distance running. Subjects participated in these activities for a minimum of five hours per week for six months. Subjects were evaluated as part of a larger long-term body composition study as described in [18] (Table 2).

#### Longitudinal Data

Phase I of the Comprehensive Assessment of Long-term Effects of Reducing Intake of Energy (CALERIE) trial tested the effects of calorie restriction on biomarkers of age-related disease [19]. Our study uses the reference database of age, height, weight, gender, and DXA body composition measurements developed from the CALERIE data at baseline, 3 months and 6 months from the Pennington Biomedical Research Center site. Twelve of the CALERIE subjects were placed on a very low-calorie diet (890 kcal/d), twelve were placed on a low-calorie diet (25% below baseline energy requirements), and twelve were prescribed a combination of caloric restriction (12.5% below baseline energy requirements) and exercise (physical activity increased to 12.5% above baseline total energy expenditures). Thirty of the subjects were overweight and six of the subjects had a baseline BMI (kg/m^2^) classifying them as obese. The average change in weight for the sixth month period was -8.8 kg.

DXA body composition measurements for seventeen female bariatric surgery patients were recorded for a two year period after surgery in [20,21]. We developed a reference database consisting of age, height, weight and body composition developed from the study in [20,21]. Measurements were obtained at baseline, 6 months, one year and two years following the surgery. The two year average change in weight for the surgery patients was -21.2 kg.

Twenty-four severely obese female gastric bypass patients were followed for a period of one year in the study described in [22]. The Siri 3-compartment model, a measurement that has been validated for use in extremely obese subjects, was used to estimate percentage body fat at baseline and follow-up. We used age, height, weight and body composition measurements at baseline and one year from this study. The average amount of weight loss for the gastric bypass subjects was -48.4 kg.

The subjects in the Minnesota Starvation Experiment (16) consisted of thirty-six white males, ages 22 to 33 years old, which were carefully selected from 400 volunteers in the Civilian Public Service for the ability to withstand long-term caloric restriction [23]. The experiment was set up into a period where baseline expenditures were determined, followed by a 24 week period of calorie reduction to approximately 1800 kcal/d. Underwater weighing body composition measurements were obtained at baseline, 12, and 24 weeks. Our reference database uses height, weight, and body composition at all three points in time. The Minnesota Starvation Experiment consists of lean individuals who lost on average 16.8 kg over the 24 week period.

The longitudinal effects of overfeeding on body composition for 22 subjects were examined in [24,25]. DXA measurements of body composition were obtained for 22 subjects, 12 of which had a BMI over 25 kg/m^2 ^and 10 who were of normal range BMI. Subjects were overfed 1000 kcal/d over baseline energy requirements for a period of 8 weeks. Average body mass gain over the 8 week period was 3.7 kg.

### Statistical and Mathematical Methods

Because any continuous function can be approximated by a polynomial, we determined the best fit polynomial with *FM *as the independent variable. We tested for statistically significant effects of powers of *FM*, *A*, *H*, race, gender, and interactions between these variables. Races considered were, white non-Hispanic, Hispanic, African American, and Asian. JMP (Release 8; SAS Inc, Cary, NC) was used for data description, statistical analysis, and analysis of variance which included paired t-tests and linear regression analysis. Analysis of variance was used to test for the effects of fat and its corresponding powers, age, height, gender and race. Numerical solutions to algebraic equations were conducted using Maple computer algebra system software (Release 12; Waterloo, CANADA) combined with Microsoft Excel (Release 2007; Seattle, WA) by using the Maple add-in feature in Excel.

## Results

### Cross Sectional NHANES and Longitudinal Weight Change Data

**Figure 1:**
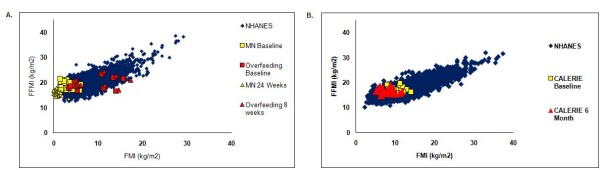
**Position of several studies on the NHANES band**. A. *FFM *index (kg/m^2^) versus *FM *index (kg/m^2^) for men from NHANES, the Minnesota Starvation Experiment (baseline and 24 week data), and overfed subjects (baseline and 8 week data) [14,15,19,23-25]. B. *FFM *index (kg/m^2^) versus *FM *index (kg/m^2^) for females from NHANES, CALERIE (baseline and 6 month data) (12,18).

**Figure 2:**
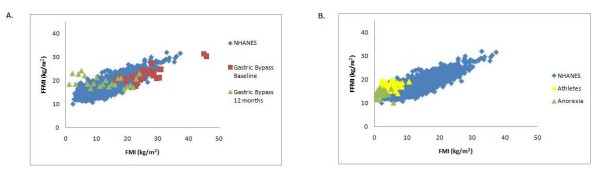
**Position of several studies on the NHANES band (females)**. A. *FFM *index (kg/m^2^) versus *FM *index (kg/m^2^) of NHANES and gastric bypass subjects at baseline and 12 months [15,22] B. *FFM *index (kg/m^2^) versus *FM *index (kg/m^2^) of NHANES, athletes, and anorexic subjects [17,18].

The cross-sectional FFMI-FMI relationship generated by the NHANES data is shown in Figure 1 and Figure 2. The NHANES male data with overlays of the data from the overfeeding study conducted by Levine [24,25] and the Minnesota Starvation Experiment ([23]), are shown in Figure 1A. Longitudinal data for females from CALERIE [19], overlay the NHANES cross-sectional FFMI-FMI plot of Figure 1B. Figure 2B consists of overlays of the anorexia nervosa patients in [17] and the athletic subjects [18] and the Figure 2A depicts gastric bypass subjects baseline and longitudinal body composition data [22].

### New *FFM *Models

Several iterations of the statistical model that considered all possible interactions between race, gender, age, height, and powers of *FM *were performed to obtain a parsimonious final model. The final model was derived by preserving higher values of the regression coefficient (*R*^2 ^> 0.86) while considering a reduced set of variables with statistically significant parameter estimates *P *< 0.001, yielding the class of fourth order polynomials in Table 3.

**Table 3:** Class of *FFM *models developed from the NHANES data

Group	*FFM*-fat Model
AA Female	*FFM *= -69 + 2.5*F *- 0.04*A *+ 0.3*H *- 0.002*FA *- 0.01*FH *- 0.047*F*^2 ^+ 0.00003*F*^2^*A *+0.0000004*F*^4 ^+ 0.0002*F*^3 ^+ 0.0003*F*^2^*H *- 0.000002*F*^3^*H*

AA Male	*FFM *= -69 + 3.6*F *- 0.04*A *+ 0.7*H *- 0.002*FA *- 0.01*FH *+ 0.00003*F*^2^*A *- 0.07*F*^2 ^+ 0.0007*F*^3 ^- 0.000002*F*^4 ^+ 0.0003*F*^2^*H *- 0.000002*FH*

NAA Female	*FFM *= -72.1 + 2.5*F *- 0.04*A *+ 0.7*H *- 0.002*A *- 0.01*FH *- 0.04*F*^2 ^+ 0.00003*F*^2^*A *+ 0.0000004*F*^4 ^+ 0.0002*F*^3 ^+ 0.0003*F*^2^*H *- 0.000002*F*^3^*H*

NAA Male	*FFM *= -71.7 + 3.6*F *- 0.04*A *+ 0.7*H *- 0.002*FA *- 0.01*FH *+ 0.00003*F*^2^*A*-0.07*F*^2 ^+ 0.0006*F*^3 ^- 0.000002*F*^4 ^+ 0.0003*F*^2^*H *- 0.000002*F*^3^*H*

*FFM *- fat models generated through a linear regression analysis of NHANES cross-sectional data that tested for the effects of age, height, race and gender, and powers of *FM*. Age in years is represented by the variable, *A*, height in cm is represented by *H*, *FM *in kg is represented by *F*, and *FFM *denotes fat free mass in kg. Separate indicator variables for all the non-African American (NAA) races were not statistically significant yielding two race indicator variables, NAA and African American (AA). The differentiated race models are listed as separate rows in the table.

Separate indicator variables for the non-African American (NAA) and non-white ethnic group were not found to be statistically significant. Hence, the non-African American (NAA) group includes all ethnic groups other than African American subjects. As a result, in the final model reduced to two separate race indicator variables, NAA and African American (AA).

### Variation in NHANES band

**Figure 3:**
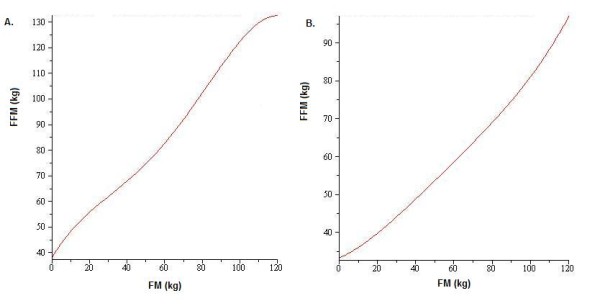
***FFM*-*FM *curves generated by NHANES**. A. An example of the *FFM *-*FM *curve generated by the model in Table 3 for males, age 30, years height 170 cm. B. An example of the FFM-FM curve generated by the model in Table 3 for females, age 30, height 163 cm. Both sample curves were generated for race NAA.

**Figure 4:**
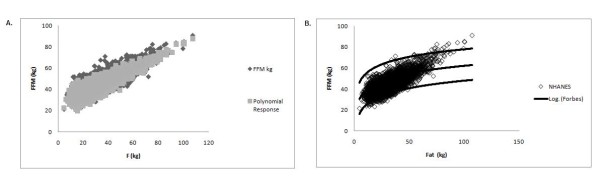
**Position of different Forbes curves on the NHANES band**. A. Female *FFM *(values (grey) obtained from substituting NHANES *FM *values [14,15] into NAA female polynomial in Table 3 overlaying NHANES actual fat free mass (black). B. Three different translates of the Forbes curve from Equation 2 overlaying NHANES actual *FFM *values for females.

The class of models in Table 3 generates different *FFM*-FM curves reflecting the variation observed in the NHANES band. Thus, fixing age, height, gender and race will yield a specific *FFM*-*FM *relationship. Figure 3 depicts plots of two sample curves; Figure 3A for non-African American males, age 30, height 170 cm and Figure 3B for non-African American females, age 30, height 163 cm. The variance due to age, height and race captures almost the entire width of the band as seen in Figure 4A. The Forbes curve acts as an averaging curve, slicing through the NHANES band. Three translates of Forbes original curve are shown in Figure 4B for females by varying the value of *D *in Equation 2. The translates of the Forbes curve indicate how the value of *D *relates to position within the NHANES band.

Of particular interest is how longitudinal data may encompass the NHANES band in the case of large weight loss. The transition from baseline to longitudinal body composition of the gastric bypass surgery patients can be observed in Figure 2A which is an overlay of the data from [22] and the NHANES FFMI-FMI relationship. The plot shows the trend of body composition for the most part follows the NHANES band. There are several conjectures of why some subjects move to the outer edges of the band which we pose in the Discussion section.

### Zero Fat

**Figure 5:**
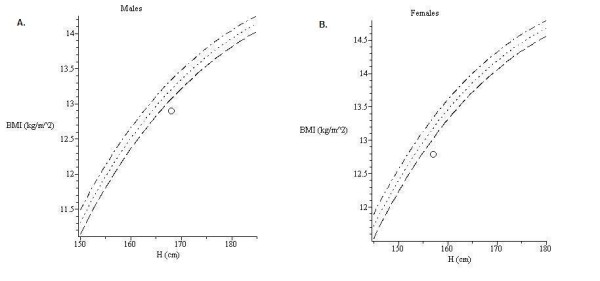
**Plots of *FFM *versus height at zero *FM***. Plots of BMI (kg/m^2^) versus height (cm) for A. males and B. females generated using the model in Table 3 after substituting *FM *equal to zero and race set to NAA. Age was set at 20 (dash-dot), 30 (dot) and 40 (dash). Circles represent mean BMI data against mean height for surviving subjects with mild edema [13].

The set of NHANES generated polynomials have a non-zero intercept and can be used to estimate the lowest theoretical BMI as a function of height. Figure 5 is a plot of level curves for males (Figure 5A) and females (Figure 5B) of BMI versus height while holding age fixed for zero fat. Age vertically translates each of the increasing functions of BMI versus height. The observation that NHANES estimate of BMI is increasing as a function of height at zero fat and that these curves are concave down can be easily proved using signs of the first and second derivative (see Supplemental Material). Mean BMI data of surviving subjects of the 1992-93 famine in Somalia with mild edema are plotted alongside the NHANES generated polynomials [13].

### NHANES Models-Forbes Model

The Forbes model was used to successfully estimate longitudinal changes in *FFM *during weight change for numerous data sets [12]. We can generate analogous estimates using the NHANES polynomial equations. These estimates are obtained by using the selected model to estimate baseline and final body composition using measured baseline weight and magnitude of weight change, thereby being able to quantify changes by subtraction of end values from baseline values. In the case of the NHANES polynomial equations, we would require age, height, gender, and race information also.

For example, if an African American female age 34, height 165 cm with baseline total body mass of 75 kg loses 10 kg of total body mass we can estimate baseline *FM *by setting total mass equal to 75 and using the relationship that total body mass is equal to the sum of *FM *and *FFM*; 75 = *FM *+ *FFM*. We then substitute the NHANES expression for an African American female, age 34, height 165 cm for *FFM*:

where the bracketed portion is *FFM *given by the NHANES polynomial. This leaves only one unknown variable, which is *FM*. We can solve for *FM *algebraically using Maple software which yields *FM *= 27.75 kg. A similar calculation with the left hand side of the equation set as 75 kg of total body mass will give us the *FM *as predicted by the NHANES polynomial equation at the changed weight; *FM *= 20.74 kg. *FFM *is obtained either from substituting the *FM *values into the polynomial or by subtracting *FM *from body mass. In this manner, we can determine the longitudinal change in *FFM*. In the above example the change in *FFM *would be -2.99 kg.

Table 4 provides the mean error between measured and predicted *FM *at baseline, final time, and total change in *FFM *over the period of the study for the NHANES and Forbes models. For a male version of a Forbes model, we generated a log linear fit to men of average stature in the NHANES dataset. Specifically, average height was found to be 174.3 ± 7.9 cm. Thus we selected all males in the data set with height ranging from 172 - 176 cm and fit a log linear curve through the data arriving at a male Forbes model (Equation 4, Table 1).

**Table 4:** Model predictions of Δ*FFM*

	NHANES		Forbes		NHANES ΔFFM	Forbes ΔFFM
	Baseline	Final	Baseline	Final		
Anorexia [17]	1.5 ± 3.1	--	2.5 ± 2.8	--	--	--

Athletes Males [18]	8.0 ± 4.5	--	9.3 ± 5.3	--	--	--

Athletes Females [18]	6.1 ± 2.9	--	3.7 ± 3.7	--	--	--

Bariatric Surgery [20,21]	-3.8 ± 3.2	-5.7 ± 3.2	1.4 ± 3.2	4.7 ± 3.3	-2.3 ± 3.0	-1.0 ± 3.2

CALERIE 6 Month Females [19]	-1.6 ± 3.2	1.2 ± 3.0	-2.0 ± 2.7	0.9 ± 2.5	-0.4 ± 1.1	-0.3 ± 1.2

CALERIE 6 Month Males [19]	4.4 ± 3.2	5.7 ± 4.7	4.9 ± 3.1	6.1 ± 4.4	-0.5 ± 1.2	-0.4 ± 1.2

Gastric Bypass [22]	2.8 ± 3.9	-2.0 ± 7.2	-5.0 ± 4.5	-2.3 ± 5.2	-7.7 ± 4.7	-0.3 ± 5.5

Overfeeding Males [24,25]	-2.2 ± 6.1	-0.5 ± 5.4	-2.2 ± 6.6	-0.3 ± 5.5	0.1 ± 1.5	-0.1 ± 1.6

Overfeeding Females [24,25]	0.2 ± 3.3	-2.0 ± 3.1	-0.6 ± 3.8	-2.7 ± 3.4	0.8 ± 1.1	0.7 ± 1.3

Minnesota [23]	2.9 ± 3.3	5.2 ± 3.0	0.6 ± 2.8	4.6 ± 2.2	2.2 ± 2.0	0.6 ± 2.1

Mean error between measured and predicted *FM *at baseline and at the end of the weight change study along with the mean error in the Δ*FFM*. The NHANES model is comparable to the Forbes model except in the case of the gastric bypass subjects.

**Figure 6:**
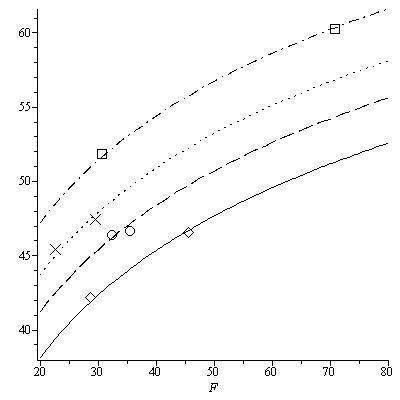
**Translations of Forbes curves calibrated to data**. Translations of Forbes models where the parameter *D *is fit to mean baseline and weight change data for gastric bypass surgery subjects (squares, dash dot curve) [22], bariatric surgery subjects (diamonds, solid curve) [20,21], CALERIE (crosses, dotted curve) [19], overfeeding (circles, dashed curve) [24,25].

Earlier studies have established that the Forbes model provides a good estimate of *FFM *change during weight change [12], even though the Forbes model may not accurately predict body composition. The reason behind this discrepancy is that although the baseline and final body composition value may not necessarily be located on the original Forbes curve, they are points on a parallel Forbes curve (Figure 6). The slope of the secant line between the points is equal to the slope of the secant line between the points on the original Forbes curve and as a result can be used to estimate the change in *FFM *effectively.

## Discussion

The class of derived *FFM *- *FM *polynomials account for most of the variation observed in the NHANES data by considering age, height, gender and race as additional covariates allowing for immediate use of individualized *FFM *curves within a one dimensional differential equation model. This allows for generation of separate curves due to individual parameters as opposed to fitting the value of *D *to specific populations in order to translate the Forbes curve.

In addition to providing a class of models that can be paired to a one dimensional energy balance differential equations, there are several interesting observations produced by the NHANES polynomials. One application of the NHANES generated models is determination of the width of the band for specific *FM *values. For example, we applied the NHANES models to determine BMI as a function of height for zero fat (Figure 5). Theoretically, these values estimate the lowest possible BMI and are supported by the close correlation to mean data collected during the 1992-93 Somalia famine in [13] also plotted in Figure 5. For the two data points, the mean age for males was 32 and the mean age for females was 35. Out of the 261 subjects in the famine study, 51 had severe edema which impacted their BMI. However, we can compare the subjects from [13] who had severe edema to the 24 week data from the Minnesota Starvation Experiment some of whom also experienced edema. The mean BMI for the famine subjects with edema was 15.4 at a height of 167 cm and the mean BMI of the Minnesota subjects at 24 weeks was 16.4 with a mean height of 179.5 cm [13,23]. Although these results are confounded by edema, it provides experimental support for the conclusion that BMI at zero fat is an increasing function of height. The polynomial regression models have several limitations. The effect of physical activity was not incorporated as a covariate. However, as observed with the placement of the athletes within the NHANES band (Figure 2B), physical activity does affect the position of the *FFM*-*FM *value on the band. Moreover, increased free-living physical activity was observed during weight gain in [24,25] and therefore can also be a factor for longitudinal body composition during weight change. It also appears that for the case of gastric bypass, changes in *FFM *for a cohort of subjects are less than estimated by the NHANES band and their resulting regression models (Figure 2A). This may be due to available active tissue within *FFM *or increased physical activity as a result of weight loss and remains to be investigated although we point out that this observation does not apply to energy balance equations that model weight change as a function of changes in energy intake and activity.

Through access of several sources of longitudinal data, we confirm the conclusions made in [12] that the original Forbes model in Equation 1 and the similarly generated male Forbes model in Equation 2, estimate the change in *FFM *during weight change with a high degree of accuracy. The Forbes model was generated as a cross-sectional fit of the relationship between *FFM *and *FM*, however, it would be incorrect to consider this as an individual curve on which baseline data begins and travels down during weight change. The Forbes model is actually a family curves where baseline and longitudinal data for an individual study are located on a translation of the original Forbes model [9] as observed in Figure 6. Because the translated curve and the original curve are parallel, the slope of the secant line between the baseline and final points on the curves are identical. Thus, the Forbes model is not a model that identifies location of a baseline point and final point during weight change, but a model that primarily quantifies the change in *FFM *as confirmed by the results in Table 4.

Discrepancies between placement of weight change subjects and normal subjects within the NHANES band is discovered by the NHANES models as observed in the massive weight loss of the gastric bypass surgery patients. The higher mean error in prediction of body composition at the changed weight and the placement of a cohort of subjects over the top of the NHANES band immediately points out that there is a distinction between subjects with the quantities of *FM *in the weight stable population and the weight change subjects (Figure 2A, Table 4). There are three possible reasons for this difference. The body composition measurements for the gastric bypass subjects were not made by DXA and thus may deviate slightly from the DXA measurements of NHANES. The second possibility is that once individuals lose great amounts of weight, they may become more active and thus increase their *FFM*. A final possibility is that the body composition of the weight changed subjects is simply different than the individuals who are already in the NHANES band. Understanding reasons behind these discrepancies will lead to improved decisions within the context of weight loss and weight re-gain.

## Conclusions

In conclusion, the relationship between *FFM *and *FM *is an integral component for differential equation energy balance models. By examining correlations of *FFM *with *FM *through the cross-sectional NHANES data and longitudinal data from calorie restriction, calorie restriction combined with exercise, bariatric surgery, gastric bypass surgery, and overfeeding studies, we established that the trend set by NHANES is traveled during weight change except in certain subjects with massive weight loss during surgery (Figure 2). Our central focus was to supply a *FFM *function of *FM *that preserves the predictive properties of the Forbes model for the change in *FFM *during weight loss, has a non-negative physically realistic intercept, and also accounts for individual variability due to age, height, race and gender. Since the development of the *FFM *formula, we have applied the new *FFM *formula within an energy balance equation with satisfactory results [26].

The resulting class of formulas satisfactorily model the large variation observed in the NHANES data and predict longitudinal data with a comparable level of accuracy to the Forbes model while also preserving non-negativity for *FFM *as *FM *tends to zero. The models predict conclusions of low fat mass data observed during famines [13] reflecting a biologically meaningful intercept. The NHANES models provide individualized estimates of baseline and longitudinal changes in body composition along with the potential to identify deviations of weight change data from the weight stable population.

These findings have implications for the future of dynamic models of human body weight change and policy regarding prescriptions for weight management.

## Competing interests

The authors declare that they have no competing interests.

## Authors' contributions

DT caried out the mathematical analysis of the data, ran the numerical simulations, and drafted the manuscript. SD supplied data and analyzed the numerical results, JAK supplied data and analyzed the numerical results, CKM supplied data and analyzed the numerical results, LM supplied data and analyzed the numerical results, AM participated in the design of the study and performed the statistical analysis, BJS supplied data and analyzed the numerical results, SBH conceived of the study and participated in its design and coordination. All authors read and approved the final manuscript.
